# Combinatorial regulation of transcription factors and microRNAs

**DOI:** 10.1186/1752-0509-4-150

**Published:** 2010-11-08

**Authors:** Naifang Su, Yufu Wang, Minping Qian, Minghua Deng

**Affiliations:** 1 LMAM, School of Mathematical Sciences, Peking University, Beijing 100871, China; 2 Center for Theoretical Biology, Peking University, Beijing 100871, China; 3 Center for Statistical Science, Peking University, Beijing 100871, China

## Abstract

**Background:**

Gene regulation is a key factor in gaining a full understanding of molecular biology. *Cis*-regulatory modules (CRMs), consisting of multiple transcription factor binding sites, have been confirmed as the main regulators in gene expression. In recent years, a novel regulator known as microRNA (miRNA) has been found to play an important role in gene regulation. Meanwhile, transcription factor and microRNA co-regulation has been widely identified. Thus, the relationships between CRMs and microRNAs have generated interest among biologists.

**Results:**

We constructed new combinatorial regulatory modules based on CRMs and miRNAs. By analyzing their effect on gene expression profiles, we found that genes targeted by both CRMs and miRNAs express in a significantly similar way. Furthermore, we constructed a regulatory network composed of CRMs, miRNAs, and their target genes. Investigating its structure, we found that the feed forward loop is a significant network motif, which plays an important role in gene regulation. In addition, we further analyzed the effect of miRNAs in embryonic cells, and we found that mir-154, as well as some other miRNAs, have significant co-regulation effect with CRMs in embryonic development.

**Conclusions:**

Based on the co-regulation of CRMs and miRNAs, we constructed a novel combinatorial regulatory network which was found to play an important role in gene regulation, particularly during embryonic development.

## Background

Gene regulation is a key factor in gaining a full understanding of molecular biology. By studying gene regulation, we uncover the mechanisms underlying gene expression, and we learn more about such biological processes as embryonic development and disease pathogenesis.

Transcription factors (TFs) compose one crucial class of regulator [1,2]. TFs exercise co-operation in their regulation by forming *cis*-regulatory modules (CRMs), which consist of multiple TF-binding sites. It has been established that most genes are controlled by CRMs, and genes targeted by the same CRM have increased similarity of expression patterns and functions [1,3,4]. As such, CRMs are the most important combinatorial regulators in gene regulation.

In the recent years, another class of regulator, microRNAs (miRNA), has also been discovered. MiRNAs are a novel class of non-coding small RNAs. They bind to the 3'-untranslated region (3'-UTR) of target transcripts and repress the translation of mRNAs or directly degrade them to regulate gene expression at the posttranscriptional level [5]. Experimental analysis has established that miRNAs have considerable effect on embryonic development, cell growth, cell death and other biological processes [6-13].

The combinatorial regulation of TFs and miRNAs has been widely identified, and it plays a major role in a variety of biological processes [6-8,12,14-16]. Because some TFs regulate the formation of miRNAs and some miRNAs affect the translation of TFs [8,12], TFs and miRNAs make up a complex regulatory network. Previous studies have investigated the structure of this network and have found that a network motif termed feed-forward loop (FFL) is a significant factor in stabilizing the gene regulation mechanism [7,9]. In the context of FFL, TF and miRNA exert effect on each other during their co-regulation. Specifically, FFLs have been found to play important roles in many biological processes, such as tumor proliferation and embryo development [12]. Experimental analysis has detected that E2F1, a well known TF that controls the cell cycle, interacts with miR-106/93/25, miR-17-92 and miR-106a-92, while, at the same time, these miRNAs silence key members of E2F1 target gene. Consequently, this FFL balances the proliferation process and plays a crucial role in proliferation [8,17].

Considering the importance of CRMs in gene regulation, we expand previous findings by developing a new combinatorial regulation paradigm which is formed by CRMs and miRNAs. We then examined the expression of genes co-regulated by CRMs and miRNAs. Meanwhile, we constructed and investigated the regulatory network composed of CRMs and miRNAs to discover the mechanism underlying their co-regulation and interaction. Furthermore, since miRNAs have been found to play a key role in embryonic development [5,12], we selected some miRNAs for detailed analysis of their effect on embryonic development.

## Results

We identified CRMs for mouse developmental genes using a computational method named MOPAT [18], and we assembled miRNAs and their target genes from TargetScanMouse [19] (See Methods).

### The effect of the combinatorial regulation of CRMs and microRNAs on gene expressions

We constructed combinatorial regulatory modules for CRMs and miRNAs. To characterize their co-regulated genes, we examined the gene co-expression patterns [2,7,18]. Mouse brain development microarray expression datasets were used for this analysis.

Previous studies have found that genes targeted by the same CRM have significantly similar expression patterns[18,20]. Such similarity is characterized by "coherence", which is defined as the mean of the Pearson coefficients between all gene pairs in the gene set. Here we characterized the CRM and miRNA combinatorial regulatory gene set in a similar manner.

**Figure 1:**
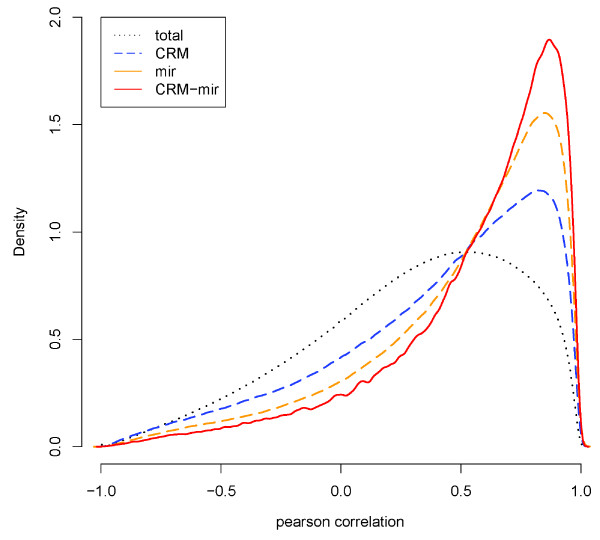
**The comparison of the correlations**. The distribution of the correlations of all gene pairs (black dotted line, mean: 0.2896930), gene pairs targeted by the same CRM (blue dashed line, mean: 0.3984559), gene pairs targeted by the same miRNA (orange dashed line, mean: 0.4927972) and gene pairs targeted by the same CRM-miRNA module (red line, mean: 0.5555378).

For every CRM and every miRNA, we constructed a new gene set consisting of genes co-regulated by the CRM and the miRNA and termed it as a CRM-miRNA module. Note that gene sets with less than two genes were eliminated. For a CRM-miRNA module, we calculated the Pearson correlations between all gene pairs. The distribution of the correlations of all CRM-miRNA modules is displayed in Figure 1. Compared with the correlations between genes targeted by the same CRM or miRNA, we found that CRM-miRNA co-regulated genes have increased similarity of expression patterns (Figure 1).

**Figure 2:**
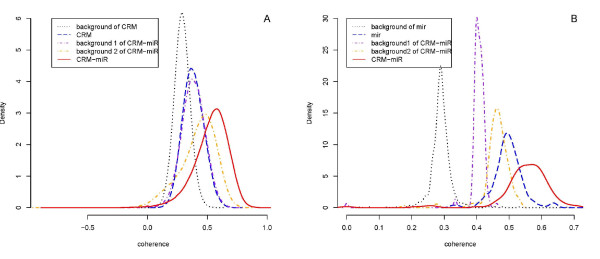
**The comparison of the coherences**. A): The distribution of the original coherences and mean coherences of all CRMs (blue, mean: 0.3827701 and red, mean: 0.5303067), as well as the coherence of CRM's background (black: 0.2896685) and CRM-miRNA's two backgrounds (purple, mean: 0.3825744 and orange, mean: 0.4197639). B): The distribution of the original coherences and mean coherences of all miRNAs (blue, mean: 0.4989906 and red, mean: 0.5589635), as well as the coherence of miRNA's backgrounds (black: 0.2900599) and CRM-miRNA's two backgrounds (purple, mean: 0.4042625 and orange, mean: 0.4989906).

Meanwhile, for each CRM-miRNA module, we took the mean of the correlations between all gene pairs as its coherence. Similarly, we obtained the coherences of all CRMs and miRNAs. To compare their coherences, we selected the corresponding CRM-miRNA modules for each CRM and found the mean of their coherence to be significantly higher than the CRM's original coherence. The mean coherences and original coherences of all CRMs are displayed in Figure2A. The comparison result of miRNA is similar (Figure 2B). In other words, in both cases, the mean coherences of CRM-miRNA modules are higher than the original coherence of either the corresponding CRM or miRNA. To evaluate the significance of the coherences, we applied randomization tests to build the background distributions (See Methods). Note that for each new module, we built two kinds of randomization sets, one randomly assigned CRM-miRNA co-regulated genes from the CRM target gene set (named as background 1), and the other randomly assigned miRNA target genes and then selects the co-regulated genes (named as background 2) (See Methods). These two backgrounds focus on the comparison of CRM-miRNA modules with their corresponding CRMs and miRNAs, respectively. We applied the same examinations on the background coherences, and the mean coherences were shown in Figure 2. The results demonstrate that the coherences of real CRM-miRNA modules are higher than the random sets. Meanwhile, we calculated the p values by repeating the tests 100 times (See Methods), and 123283 (3.05%) CRM-miRNA modules were found to have significant p values (See Additional File 1).

The results showed that CRM and miRNA co-regulated genes have significantly similar expression patterns. Thus, it follows that CRM-miRNA modules, as a combinatorial construct, can provide more insight into gene variation, inspiring us to further investigate the regulatory network formed by such modules.

### Analysis of regulatory network

Based on the above findings, we further investigated the inherent mechanism underlying CRM-miRNA modules. Similar to previous studies [7-9], we constructed a gene regulatory network consisting of CRMs, miRNAs, and their target genes. Here a CRM was predicted to target a miRNA if it had binding sites upstream of the pri-miRNA. Meanwhile, a miRNA was considered to regulate a CRM if it targeted at least one TF in the CRM (See Methods).

We further intensively investigated the structure of the network. Ten combinatorial regulation patterns have been discovered (Table 1). We measured their significance by constructing 100 random networks and calculating a Z-score for each pattern (See Methods) [9].

**Table 1:** Ten patterns in the combinatorial regulatory network.

No.	Pattern	Number of occurrences	Z-score
1		1023429	6.8716

2		14737	6.443

3		338490907	4.502

4		1359	3.944

5		401659	3.779

6		12559	-4.774

7		16663892	-5.158

8		101238	-5.467

9		179451	-8.429

10		23292181	-9.801

The first five rows are the patterns with high Z-scores.

There are five significant patterns in the regulatory network (Table 1). They show that miRNAs and CRMs tend to interact during their regulatory process. CRMs control the regulation of miRNAs (Pattern 3 and Pattern 5), but miRNAs also affect the regulation of CRMs (Pattern 2). Meanwhile, CRMs and miRNAs interact in their co-regulation of target genes (Pattern 1 and Pattern 4). The last network motifs are typical FFL, which have been found in a wide variety of biological networks. These network patterns show that genes are generally affected by multiple direct or indirect regulators, and the presence of FFL acts to minimize excessive fluctuations in gene expression, thus contributing to the stability of the whole biological system and robustness against noise [8,17].

In addition, previous studies have reported that some miRNAs locate in a gene's intron [21]. Therefore, we further examined host genes of miRNAs, and for a majority of miRNAs, we found that CRMs regulated both miRNA and host gene (See Methods). This strongly suggests that a CRM may simultaneously regulate a gene and activate a miRNA in its intron. Since this miRNA regulates its host gene and forms an FFL, the whole biological process is stabilized.

Such network performances support for our previous findings of gene co-expression and display the inherent mechanisms underlying CRM-miRNA combinatorial regulation.

### Examples related to embryonic development

#### mir-154

We selected some miRNAs for a detailed analysis. Previous studies demonstrated that mir-154 enriches in embryonic tissues and is related to embryonic development [22]. Therefore, we focused on mir-154 for further investigation.

**Figure 3:**
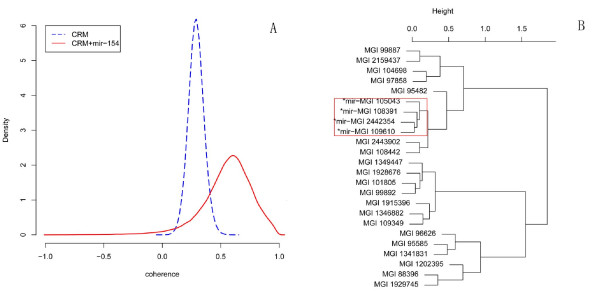
**Analysis of mir-154**. A) The distribution of coherences corresponding to CRM (blue dashed line, mean: 0.2896425) and CRM-mir-154 module (red solid line, mean 0.5547383). B) The cluster of the genes regulated by the 15374-th module. Genes targeted by mir-154 were annotated with "*mir-" before gene names.

The co-regulated gene sets formed by mir-154 and CRMs have higher coherences (Figure 3A). This finding demonstrates that genes targeted by CRMs and mir-154 have similar expression patterns, further confirming that mir-154 plays an important role in embryonic development.

We then selected the CRM consisting of M00277, M00720, M00977 for more intensive examination. Based on clustering data (the distance between two genes was defined as one minus their correlation), we found that genes targeted by mir-154 were apparently classified in the same subset (Figure 3B, marked in the red frame).

#### TCf3

There are four core TFs in embryonic cells: Sox2, Tcf3, Oct4 and Nanog [12]. We further explored their combinatorial regulation with miRNAs. Since the results were similar, we use Tcf3 as our example.

We examined gene expressions in mouse embryo cells before and after Tcf3 depletion [23]. The log ratio of gene expression after Tcf3 depletion and before treatment was used to measure the expression changing level, and we modeled this level as a linear combination of regulatory effects of Tcf3 and miRNAs[16]. A feature-selection procedure was performed (See Methods), and we finally selected 88 miRNAs having significant co-regulatory effect with Tcf3. Nine of them were verified to be targeted by the CRMs containing Tcf3 by our previous prediction as well as ChIP-seq analysis [12]. They are mir-7, mir-17, mir-25, mir-33, mir-124, mir-125, mir-128, mir-143 and mir-135. All these miRNAs form FFLs with the CRMs containing Tcf3 (as in Pattern 1 and Pattern 4; see Table 1). These findings provided us with further evidence that miRNA play an important role in embryonic development.

## Discussion and Conclusions

We have developed a new type of combinatorial regulatory module, which is formed by CRMs and miRNAs. Based on our investigation of gene expressions using this paradigm, we found that genes co-regulated by this CRM-miRNA module have more similar expression patterns than the genes regulated by either the corresponding CRM or miRNA. It verified that miRNA could buffering gene expression noise [24].

Further investigation led to the discovery of a gene regulatory network consisting of several network motifs, including FFL, which has been found to be essential in a variety of gene networks. The FFLs of CRMs and miRNAs play an important role in many biological processes, including embryonic development [8,12]. As a result of our assessments, we have gained further insight into the structure of CRM-miRNA combinatorial regulation, as well as the gene regulatory network in which these elements interact.

Moreover, since miRNAs were previously demonstrated to affect embryonic development [8,17], we selected some miRNAs related to embryonic development for further analysis. The performance of mir-154 and other miRNAs gave us more insight into the effect of CRM-miRNA co-regulation on embryonic development. Meanwhile, we applied a linear model to characterize in detail the effect of TF and miRNA. It provides more evidence to show that CRMs and the miRNAs have co-regulation.

Generally, our study sheds light on the importance of CRM-miRNA combinatorial regulation, which we demonstrated to be more powerful than either CRM or miRNA alone in gene regulation. Furthermore, we found that CRMs and miRNAs are likely to form FFL inside their network structure, helping us to gain further understanding of gene regulation.

## Methods

### Expression data

We selected seven gene expression datasets from the GEO database [25]. They are time series datasets in the embryonic development of seven different tissues in mouse, including liver development (GSE13149), eye development (GSE13103), lung development (GSE11539), brain development (GSE8091), cardiac development (GSE5671), testis development (GSE6881) and ovary development (GSE6882). We directly used their "series matrix" in GEO to calculate the Pearson correlations between gene pairs. Note that a gene may have several probesets. For a gene pair, we took the maximal correlation of their probeset pairs. Here we concentrated on the brain development data.

In addition, we extracted experimental gene expression data of Tcf3 (GSE16375) [23]. These data depict gene expressions before and after the silence of Tcf3 by doxycycline treatment. We took the mean expressions for different replicates.

### Assign CRM and microRNA target genes

We used MOPAT[18], a motif pair tree method, to predict 144490 CRMs, consisting of 494 motifs for 5355 mouse development genes.

MiRNAs and their predicted target genes were assembled from TargetScanMouse (release 5.1) [19]. Since miRNAs in the same miRNA family are similar in regulation, all miRNAs were classified into miRNA families using the annotation in miRBase [26]. In all, we analyzed 135 miRNA families and 8811 target genes. For simplicity, we shortened "miRNA family" to "miRNA" in the article.

All gene symbols were converted to MGI Marker Accession ID using the MGI database [27].

### Evaluation of the significance of coherence

To assign the significance of the CRM-miRNA co-regulated gene set, we performed a randomization test. For each CRM-miRNA target gene set, we randomly assigned its co-regulated genes from all CRM target genes and kept the size of the gene set. Meanwhile, for the randomization of CRMs and miRNAs, we randomly assign their target genes from the whole gene set. Note that for CRM-miRNA module, genes were not picked up from the whole gene set since we wanted to exclude the influence of CRM and focus exclusively on the effect of miRNAs.

We repeated the test five times to form the background distribution. Moreover, we used 100 tests to calculate the p-value, defined as the proportion of random sets that had the same, or higher, coherence than the real set. We have considered multiple testing problems and have corrected the p values using FDR modification.

Meanwhile, to directly evaluate the effects of miRNAs, we generated some artificial miRNAs by randomly assigning their target genes from the target genes of all miRNAs. Here we kept the distribution of the miRNAs' target gene numbers [28]. This randomization test was performed five times to form the background distribution.

Applying these two kinds of randomizations, we could construct two backgrounds and evaluate the significance of every CRM-miRNA module.

### Construction of the combinatorial regulation network

#### CRM **→** miRNA

The pri-miRNAs and their location in the genome were downloaded from miRBase [26]. Their upstream 5kb sequences were extracted from Ensembl [29].

We first applied the same approach as MOPAT [18] to search the TF binding sites in the pri-miRNA's upstream region. We extracted the position weight matrices from TRANSFAC 9.2 [30] and calculated the log-likelihood ratio scores on every site of the pri-miRNA upstream sequence. For every motif, the site with a score larger than the cutoff was predicted to be a binding site. Here the cutoff was calculated as the 99.99% quartile of a random sequence.

A CRM was predicted to regulate the miRNA if its containing motifs were all identified to have binding sites in the pri-miRNA's upstream sequence and these binding sites were within 200bp.

#### miRNA **→** CRM

All mouse position weight matrixs in TRANSFAC were mapped to the genes encoding the TFs that bind these PWMs [8]. A miRNA was predicted to target a CRM if at least one TF in the CRM had corresponding genes targeted by the miRNA.

In all, we identified 695432 CRM → miRNA pairs and 5060 miRNA → CRM pairs.

### Evaluation of the significance of patterns in the regulation network

We constructed 100 random networks to measure the significance of every regulatory pattern by swapping the edges of the real network randomly. Thus, each random network had the same number of CRMs, miRNAs and genes with the actual network. We also kept the same number of CRM-gene, miRNA-gene and CRM-miRNA interaction pairs, respectively. For every pattern, a z-score was calculated as the difference of its actual occurrence and the average of its random occurrences, normalized by the standard deviation of the random occurrences. Patterns with high z-score were supposed to be enriched in the regulatory network [9].

### Host genes

We extracted sixteen miRNAs that have host genes from miRBase [26]. After overlapping with our mouse development gene of interest, there remained ten miRNAs, while nine of them were targeted by the same CRMs with its host genes.

### Analysis of the experimental data of Tcf3

The relationship between gene expressions and the regulatory effects of Tcf3 and miRNAs can be formulated as a linear model [16]:

(1) $$g_{k}=c+a_{TF,k}b_{TF,k}+\sum_{i=1}^{n}a_{mir_{i},k}b_{mir_{i},k}+error$$

*g*_*k *_is the log ratio of expression of the k-th gene after Tcf3 depletion and before treatment; *b*_*TF,k *_and $b_{mir_{i},k}$ are 1 or 0 to indicate whether the k-th gene is targeted by Tcf3 or the i-th miRNA. The parameters *a*_*TF,k *_and $a_{mir_{i},k}$ show the regulatory effects of Tcf3 and the i-th miRNA; in other words, they measure the expression variation of genes targeted by Tcf3 and the i-th miRNA.

To select miRNAs in this model, we first used an alternative model, considering Tcf3 and one miRNA at one time, that is

$$g_{k}=c+a_{TF,k}b_{TF,k}+a_{mir_{i},k}b_{mir_{i},k}+error$$

All miRNAs were fitted to this model, and those with significant p value were selected to fit the original model (1). We used a stepwise linear regression of AIC criteria to select miRNAs. For the selected miRNAs, their target genes had significantly different expression levels after Tcf3 depletion, indicating that these miRNAs have significant interaction with Tcf3.

## Authors' contributions

MPQ and MHD proposed the problem and directed the overall direction of the work. NFS extracted the data and analyzed the expression of the co-regulated genes. YFW constructed the regulatory network of CRM and miRNAs and examined its structures. NFS drafted the manuscript and all authors read and approved the final manuscript.

## Supplementary Material

Additional file 1 **significant CRM-miRNA pairs and their p values**.This table contains the co-regulated CRM-miRNA pairs with p values less than 0.05. P values are adjusted by FDR modifications. Click here for file
